# *In vitro* Lipolysis and Leptin Production of Elephant Seal Blubber Using Precision-Cut Adipose Tissue Slices

**DOI:** 10.3389/fphys.2020.615784

**Published:** 2020-12-10

**Authors:** Cathy Debier, Laura Pirard, Marie Verhaegen, Caroline Rzucidlo, Gilles Tinant, Clément Dewulf, Yvan Larondelle, Donald R. Smith, Jean-François Rees, Daniel E. Crocker

**Affiliations:** ^1^Louvain Institute of Biomolecular Science and Technology, UCLouvain, Louvain-la-Neuve, Belgium; ^2^Department of Biology, Sonoma State University, Rohnert Park, CA, United States; ^3^Department of Microbiology and Environmental Toxicology, University of California, Santa Cruz, Santa Cruz, CA, United States

**Keywords:** precision-cut slices, adipose tissue, elephant seal, *in vitro* culture, lipolysis, glycerol, leptin, marine mammal

## Abstract

Adipose tissue plays key roles in energy homeostasis. Understanding its metabolism and regulation is essential to predict the impact of environmental changes on wildlife health, especially in fasting-adapted species. However, *in vivo* experimental work in wild vertebrates can be challenging. We have developed a novel *in vitro* approach of precision-cut adipose tissue slices from northern elephant seal (*Mirounga angustirostris*) as a complementary approach to whole animal models. Blubber biopsies were collected from 14 pups during early and late post-weaning fast (Año Nuevo, CA, United States), precision-cut into 1 mm thick slices and maintained in culture at 37°C for at least 63 h. The slices exhibited an efficient response to ß-adrenergic stimulation, even after 2 days of culture, revealing good *in vitro* tissue function. The response to lipolytic stimulus did not vary between regions of outer and inner blubber, but was higher at early than at late fast for inner blubber slices. At early fast, lipolysis significantly reduced leptin production. At this stage, inner blubber slices were also more efficient at producing leptin than outer blubber slices, especially in the non-lipolytic condition. This model will aid the study of adipose tissue metabolism and its response to environmental stressors in marine mammals.

## Introduction

Adipose tissue has been traditionally considered for its role in insulation and energy storage. More recently, it has been shown to be an essential endocrine organ, secreting adipokines that are involved in various crucial biological functions, such as appetite regulation, immunity, inflammation, and regulation of glucose and lipid metabolism (Frühbeck et al., 2001; Coelho et al., 2013). It is also an important tissue in the storage and endogenous release of many lipophilic environmental pollutants (Lee et al., 2017).

In marine mammals, sub-cutaneous adipose tissue (blubber) is a multi-functional organ playing a key role in metabolic homeostasis and energy balance regulation, especially in fasting adapted species such as northern elephant seals (NES – *Mirounga angustirostris*) (Strandberg et al., 2008). Knowledge of adipose tissue metabolism is essential to understand the impacts of environmental factors (pollution, decrease of food quantity and quality, disturbances linked to sonar, oil drilling, boat traffic and coastal development) on adipose tissue function (Khudyakov et al., 2017), and more generally on health and survival of the organism.

Studying adipose tissue metabolism and its response to environmental stressors in wild free-ranging aquatic organisms, while important, is also challenging for obvious practical and ethical reasons. *In vitro* approaches, when properly developed and executed, may provide an important and viable complement to whole animal models by minimizing the animal impact, and allowing experiments with multiple treatments under controlled conditions. Marine mammal blubber is relatively accessible to sample. In fact, for some species such as large cetaceans, it is the only tissue that is feasible to sample in living individuals (Hunt et al., 2013). If adipocyte cell culture models are well established for laboratory rodent species (Bourez et al., 2013; Louis et al., 2014c,d), such systems have only scarcely been attempted for marine mammals (Louis et al., 2014b; Routti et al., 2016). We previously isolated and cultured adipocyte precursor cells from NES (Louis et al., 2014b). While promising results were obtained, the system needed further improvement to obtain efficient and consistent *in vitro* adipocyte differentiation.

Cultures of tissue explants represent an alternative to cell cultures as they are simpler to prepare and close to the *in vivo* reality in terms of cell environment, diversity and tissue architecture. The use of explants has been recently developed with gray seal (*Halichoerus grypus*) blubber (Bennett et al., 2017) to assess the effects of environmental pollutants such as polychlorinated biphenyls (Robinson et al., 2018). Besides explants, precision-cut tissue slices enable the production of multiple reproducible and standardized tissue samples that are thin enough to allow diffusion of substrates, while ensuring comparisons between conditions (de Kanter et al., 2005). Precision-cut tissue slices mimic the multi-cellular complexity, extracellular interactions and structural and functional features of the whole organ. Precision-cut tissue culture methods are commonly used for tissues such as liver, kidney, intestine or lung (human, rodent, fish) (e.g., Wohlsen et al., 2003; De Graaf et al., 2010; Lemaire et al., 2012, 2016; Stribos et al., 2016; Thiele et al., 2017). On the other hand, the method appears much less developed for adipose tissue, which might be due in part to the technical difficulties linked to its texture. Very recently, Schopow et al. (2020) successfully established a slice culture system from human adipose tissue to examine cellular mechanisms involved in obesity, highlighting the interest of this promising tool to study the complex processes underlying adipose function and dysfunction. Such a model however, is lacking for wild animals such as marine mammals.

To address this gap, we sought to develop a precision-cut adipose tissue slice (PCATS) model from NES. Because of its accessibility, physiology and natural life history, the NES represents an excellent model to investigate adipose tissue metabolism (Khudyakov et al., 2017). This physiologically obese marine mammal is characterized by a thick layer of blubber and is relatively easy to handle and sample during its periods on land (breeding, molting, post-weaning development). NES undergo extended multi-month fasting periods while on land, during which they rely on their fat stores to meet their energy requirements (Houser et al., 2013; Crocker et al., 2014). The NES colony of Año Nuevo, CA, United States, is situated near the University of California at Santa Cruz, which enables the rapid transfer of live tissue from the colony to the laboratory for experiments. Blubber biopsies were collected from free-ranging NES weaned pups. After their 27-day suckling period, NES pups are abruptly weaned and initiate a fasting period for ∼10 weeks during which they develop and acquire the capabilities to swim and dive. Blubber biopsies were precisely cut into 1 mm thick slices that were kept alive in culture media at 37°C. Tissue slices function was closely monitored using assessments of elementary metabolic function of adipose tissue, such as induced lipolysis and secretion of the adipokine leptin. Lipolysis was triggered with isoproterenol, a synthetic agonist of ß-adrenergic receptors. Experiments were conducted using tissues collected from pups early (within 1 week of feeding) and late (extended fasting state) in their post-weaning fast in order to compare the function of the tissue between these two very different physiological states. We also investigated whether slice response to isoproterenol or production of leptin differed according to blubber depth. Indeed, inner blubber (closer to the muscle) is usually considered as the layer from which lipids are mainly mobilized during periods of energy deprivation while outer blubber is deemed as more stable (Best et al., 2003; Strandberg et al., 2008).

## Materials and Methods

### Animal Capture and Sample Collection

All methods were carried out in accordance with relevant guidelines and regulations. All capture and handling procedures were approved by the Sonoma State University Institutional Animal Care and Use Committee (IACUC #2017-56) and were performed under the National Marine Fisheries Service Marine Mammal Permit #19108. Free ranging weaned NES pups were sampled at Año Nuevo State Reserve, CA, United States (37°06′30″N, 122°20′10″W), from February to May 2018. Four early weaned pups were sampled for PCATS method setup. Afterward, six early (three males and three females – within the first 10 days after weaning) and eight late (four males and four females – after 2 months of fast) weaned pups were sampled for lipolysis experiments. Pup anesthesia was initiated with an intramuscular injection of Telazol (1 mL/100 kg of estimated body mass) (Ketaset, Fort Dodge Animal Health, Fort Dodge, IA, United States) and then maintained with an intravenous injection of Ketamine (as needed) (Ketaset). Full-length blubber biopsies were taken in the lateral pelvis area with 6-mm biopsy punches (Uni-Punch, Premier, Plymouth, MA, United States). Samples were rinsed in phosphate buffered saline (Life Technologies, Thermo Fisher Scientific, Waltham, MA, United States) before being kept in sterile sampling medium at around 25°C as recommended for adipose tissue explants (Fried and Moustaid-Moussa, 2001; Bennett et al., 2017), in order to slow down the metabolism without temperature-shocking the tissue. The sampling medium, which was preliminarily oxygenated, was adapted from Bourez et al. (2013) and Louis et al. (2014c,d) and contained Dulbecco’s modified eagle medium (DMEM) 4,5 g/L of glucose (Gibco, Life Technologies) supplemented with 10 mM HEPES buffer solution (Life Technologies), antibiotic and antifungal mixture (100 U/mL penicillin, 100 U/mL streptomycin, 250 ng/mL amphotericin (Life Technologies), 1 mg/mL gentamycin (Life Technologies), 40 U/mL nystatin (Sigma-Aldrich, Saint-Louis, MO, United States)) and 2 mM Glutamax (Life Technologies) (Bourez et al., 2013; Louis et al., 2014c,d).

### PCATS Preparation and Culture

**FIGURE 1:**
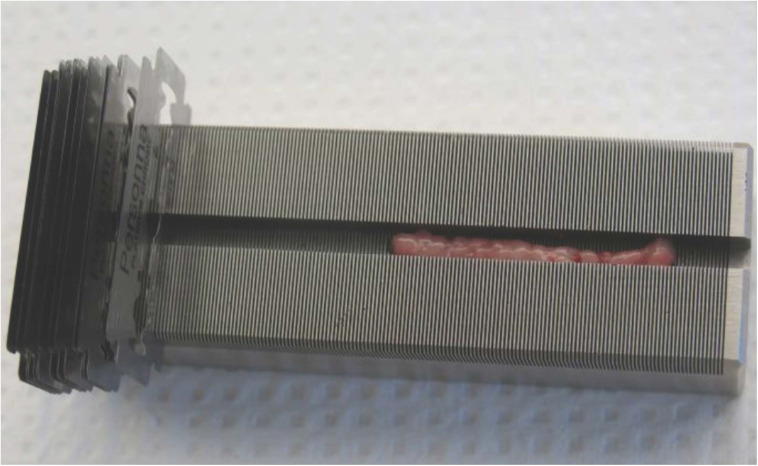
Photo of the compact device used to prepare the slices (Ted Pella Inc., Redding, CA, United States) with a blubber biopsy obtained with a 6-mm biopsy punch placed within the groove. Slices of 1-mm depth were made on small portions of the biopsy at a time with razor blades placed in their slots.

Tissues were transported to the laboratory and processed within 2–4 h after sampling. Biopsies were first split into three equal parts according to previous studies on vitamins, fatty acid and pollutant profiles (Debier et al., 2012; Louis et al., 2014a, 2016). Small portions of the inner (closer to the muscle) and outer (closer to the skin) layers were then processed under sterile conditions to produce PCATS, using a compact device built for making thin slices of rat spinal cord (Mouse and Rat Spinal Cord Matrices, Ted Pella Inc., Redding, CA, United States). The device is made of stainless steel and contains a groove that is 4 mm wide, 96 mm long and 5 mm deep (Figure 1). Preliminary tests conducted with 4 mm and 6 mm biopsy punches revealed that 6-mm biopsy punches produced blubber biopsies that best matched the groove width. Thin slices were made by placing razor blades in the slots every 1 mm. Portions of four slices were weighed in pre-weighed tubes containing 1 mL of pre-warmed sterile culture medium (same composition as the sampling medium, except that the gentamycin concentration was reduced to 50 μg/mL, no HEPES was added and the medium contained 10% (v:v) fetal bovine serum (FBS) (Gibco, Life Technologies)) in order to precisely standardize the results per unit of tissue wet weight. After weighing, slices and media were transferred in 24-well culture plates (four slices and 1 mL of medium per well) and placed in an incubator (37°C, 5% of CO_2_) on a shaker for 3 h. This first 3-h incubation period was added in order to collect and remove cellular components resulting from damaged cells at the edges of the slices. After 3 h of incubation, media were removed and replaced by new media, differing in their composition as described below. Shaking was used throughout the culture to keep a homogeneous solution, facilitate gas exchange (de Kanter et al., 2005) and avoid slice clumping.

### Lipolytic Treatments

**FIGURE 2:**
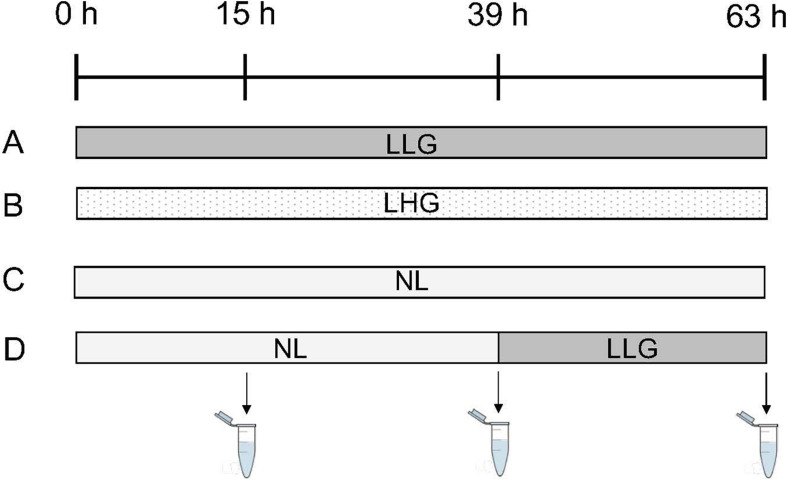
Lipolytic treatments. Four different conditions were tested: **(A)** Lipolytic condition with a low glucose concentration “Lipolysis Low Glucose (LLG)” (culture medium with 1 g/L glucose, 5% (v:v) fetal bovine serum, antibiotic and antifungal mixture, 1 μM isoproterenol and 2% (w:v) albumin), **(B)** Lipolytic condition with a high glucose concentration “Lipolysis High Glucose (LHG)” (culture medium with 4.5 g/L glucose, 5% (v:v) fetal bovine serum, antibiotic and antifungal mixture, 1 μM isoproterenol and 2% (w:v) albumin), **(C)** Non-lipolytic condition (“No Lipolysis (NL)”) (culture medium with 4.5 g/L glucose, 10% (v:v) fetal bovine serum, antibiotic and antifungal mixture, 2 mM Glutamax and 100 pg/mL insulin), and **(D)** Non-lipolytic condition (NL) followed by lipolysis (LLG) after 39 h of culture. The different media were collected and replaced by fresh media after 15 and 39 h of culture. Media were finally harvested after 63 h of culture.

Four different conditions were tested (Figure 2). The first two conditions referred to two lipolytic treatments, differing in their glucose concentration (“Lipolysis Low Glucose” (LLG) and “Lipolysis High Glucose” (LHG)) with DMEM 1g/L or 4.5 g/L glucose, 5% (v:v) of FBS, antibiotic and antifungal mixture (100 U/mL penicillin, 100 U/mL streptomycin, 250 ng/mL amphotericin, 50 μg/mL gentamycin, 40 U/mL nystatin), 1 μM isoproterenol (Sigma-Aldrich) and 2% (w:v) bovine albumin (Bovine serum albumin, BSA) (Sigma-Aldrich). The concentrations of isoproterenol and albumin were chosen according to Harmancey et al. (2005) and Louis et al. (2014c,d). The third condition (“No Lipolysis” (NL)) simulated a non-lipolytic state and was composed of DMEM (4.5 g/L glucose), 10% (v:v) FBS, antibiotic and antifungal mixture (100 U/mL penicillin, 100 U/mL streptomycin, 250 ng/mL amphotericin, 50 μg/mL gentamycin, 40 U/mL nystatin), 2 mM Glutamax and 100 pg/mL insulin (Sigma-Aldrich). The fourth condition referred to a non-lipolytic state (NL) followed by lipolysis (LLG) after 39 h of culture. The different media were collected and replaced by fresh media after 15 and 39 h of culture. Media were finally harvested after 63 h of culture. This timing was chosen according to preliminary experiments, which showed that, beyond this point, cumulative glycerol release tended to reach a plateau for the conditions in which lipolysis was induced since the beginning of the culture (Debier, unpublished results). The insulin concentration in FBS was 237 pg/mL. By adding 5% of serum in the LLG and LHG conditions, the final insulin concentration in the medium was 11.9 pg/mL. Insulin was added in the NL medium in order to obtain a final insulin concentration of 123.8 pg/mL (23.8 pg/mL from the 10% of FBS and 100 pg/mL from the insulin directly added to the medium). The final concentration of glucose was 0.9 g/L in LLG and LHG media and 4.2 g/L in NL medium.

### Tissue Viability

Tissue viability was assessed during two different cultures (two different pups) for the three conditions investigated (NL, LLG, LHG) by measuring the release of lactate dehydrogenase (LDH) in the culture medium and compare it to the LDH present in the tissues, as reviewed in de Kanter et al. (2005). The activity of LDH was determined using the cytotoxicity detection kit (Cytotoxicity Detection Kit (LDH), Roche, Sigma-Aldrich). Before the beginning of the culture as well as at the end of it, tissue slices were lysed with 1% Triton X-100 (Sigma-Aldrich) to serve as positive control (activity of LDH present within the tissues before and at the end of the culture). Before initiation of the experiment with the different lipolytic conditions (NL, LLG, LHG), the freshly obtained slices remained in the same culture medium (NL) during 3 h. The amount of LDH released in the culture medium during that period (corresponding to the cells damaged by the slicing itself) was higher (20%) because of the loss of enzymes from the damaged cells on the cutting edges. This higher enzyme leakage was also reported in the review of de Kanter et al. (2005) on precision-cut organ slices. The LDH leakage after 3 h was subtracted from the LDH present in the slices before the start of the culture (corresponding to damaged and non-damaged cells) in order to obtain an LDH value corresponding to the non-damaged cells (assigned as “LDH initial”). The LDH released in the culture medium between *T = 0* and *T = 15* h and between *T = 15* and *T = 39* h was compared to “LDH initial” to calculate a percentage of mortality. For the last medium harvesting, the LDH released in the culture medium (between *T* = 39 and *T* = 63 h) was compared to the LDH activity present in the slices at the end of the experiment.

### Lipolysis Measurements

Lipolysis was monitored by quantifying glycerol in culture media at four different times (*T* = 0 h which corresponds to the start of the lipolytic treatments, after the 3 h of incubation to collect damaged cells contents, *T* = 15, 39, 63 h). Glycerol was measured using a glycerol assay kit (MAK117-AKT, Sigma-Aldrich) and Perkin Elmer Victor X3 spectrophotometer. Concentrations were calculated as μmoles per g of tissue.

### Leptin Secretion

Leptin concentrations into the culture medium were monitored at *T* = 15, 39, 63 h in the NL, LLG and LHG conditions of four of the early weaned pups and six of the late weaned pups. Leptin concentrations were measured in duplicate using a radioimmunoassay (multispecies kit, Linco, St. Charles, MO, United States) previously validated for NES (Ortiz et al., 2001). The intra-assay and inter-assay coefficients of variation were 3.43% and 5.12%, respectively.

### Histological Sections

Histological sections were prepared in subsets of adipose tissue slices. After 63 h of culture, slices from LLG and NL conditions were fixed in 4% formaldehyde before being embedded in paraffin. Five-micrometer-thick sections were processed and stained with hematoxylin and eosin (Sigma-Aldrich, Bornem, Belgium).

### Statistical Analyses

Statistical analyses were performed using JMP PRO 13.0 software.

#### Changes of Glycerol or Leptin Concentrations With Time (h of Culture) or Treatments

Distribution normality was tested using Shapiro-Wilk’s test and, when necessary, data were transformed in log_10_ to achieve normality. *T* = 0 h was not taken into account. Variance homogeneity was tested using Levene’s test. Differences between treatments within a particular time or over time within a particular treatment were assessed with linear mixed models (with the pup as a random variable and the time or treatment as fixed variables) and a *post hoc* Tukey’s test.

#### Differences of Total Glycerol or Leptin Released Over the 63 h of Culture According to Sex, Blubber Layer, or Fasting Stage

Distribution normality was tested using Shapiro-Wilk’s test and, when necessary, data were transformed in log_10_ to achieve normality. Variance homogeneity was tested using Levene’s test. Afterward, a Student *t*-test (paired data for comparisons between inner and outer blubber and non-paired data for comparisons between early and late fast or between sexes) was applied to compare the means (comparison by treatment).

The level of statistical significance was set at *p* < 0.05 for all analyses.

## Results

### Slices Description

**FIGURE 3:**
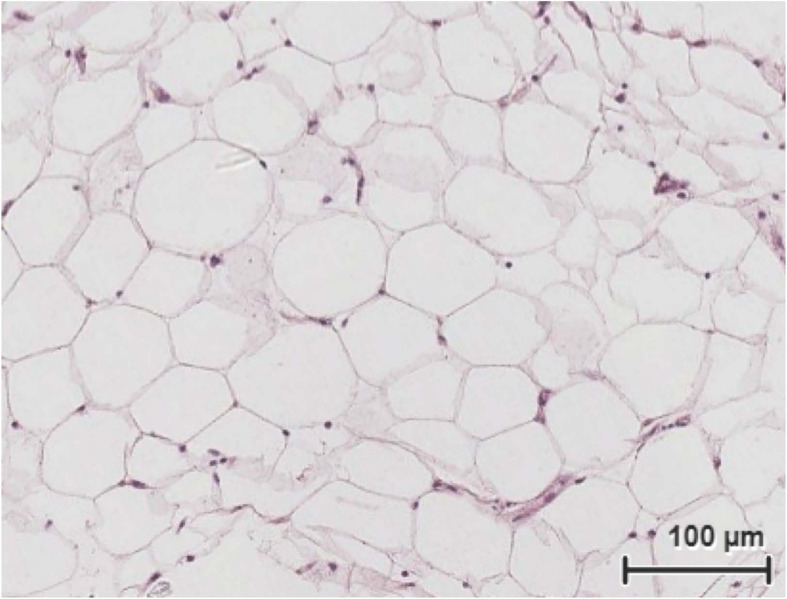
Histological sections and hematoxylin and eosin staining of northern elephant seal inner blubber slices after 63 h of culture under the lipolysis low glucose condition.

Slices of 1 mm thick and 6 mm of diameter were obtained with a portable stainless steel device (Figure 1). PCATS mean weight was 4.75 ± 1.49 mg (mean ± SD) (*n* = 111). Histological sections were conducted on inner blubber slices from late fasting pups after 63 h of culture. Round adipocytes could be observed, with their nucleus pushed to the side of the cell (Figure 3). Other types of cells, corresponding to either connective tissue, muscle or blood vessel, could also be observed. One millimeter thick slices corresponded to about 15 layers of cells.

### Viability

Four conditions were tested: one “control” condition without isoproterenol and albumin (“No Lipolysis” or NL), two conditions of ß-adrenergic-induced lipolysis (isoproterenol + albumin) with either high (4.2 g/L) or low (0.9 g/L) glucose concentrations in the medium (“Lipolysis high glucose” LHG and “Lipolysis low glucose” LLG), and a last condition in which the slices were in NL medium between 0 and 39 h and then in LLG medium from 39 to 63 h (NL LLG). Cell viability (measurement of lactate dehydrogenase release in culture medium) was maintained during the 15 h-period as cell death estimates were only 3.2 ± 3.8% for the LLG condition, 2.9 ± 3.0% for LHG condition and 0.8 ± 0.4% for NL condition during that period. Between *T* = 15 h and *T* = 39 h, it remained low at 1.1 ± 0.7% for LLG, 2.2 ± 0.6% for LHG and 1.9 ± 1.6% for NL. During the period from *T* = 39 h to *T* = 63 h, mortality was 3.6 ± 4.9% for LLG, 8.9 ± 2.4% for LHG and 6.9 ± 2.3% for NL.

### Response to Lipolytic Stimuli

**FIGURE 4:**
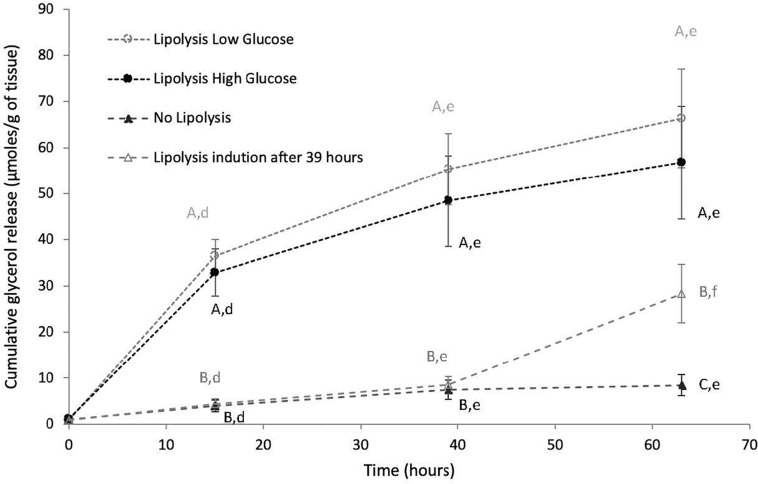
Cumulative glycerol release over time from inner blubber slices of early weaned northern elephant seal pups. Slices were cultured during 63 h in four different conditions: “Lipolysis Low Glucose,” “Lipolysis High Glucose,” “No Lipolysis,” “Lipolysis induction after 39 h.” Glycerol concentrations were normalized per unit of initial tissue wet weight (before starting the culture). Results are expressed as mean of six pups ± SEM. Differences between treatments within a particular time or over time within a particular treatment were assessed with linear mixed models and Tukey’s tests. Values with different capital letters A, B, C are significantly different (*p* < 0.05) within a precise time between treatments. Values with different lowercase letters d, e, f are significantly different (*p* < 0.05) within a precise treatment over time. Same patterns were observed for inner blubber at late fast as well as for outer blubber, at early and late fast.

Glycerol release over time in culture media was analyzed in inner and outer blubber at early and late fast. Cumulative glycerol release in culture media increased significantly over time in LLG and LHG conditions (*p* < 0.05), whereas it remained constant in the NL condition (Figure 4). As a result, glycerol levels in culture media were significantly higher in LLG and LHG conditions as compared to NL condition, from *T* = 15 h to *T* = 63 h (*p* < 0.05). Within the two lipolytic conditions, there was no significant difference of glycerol release under low versus high glucose concentration conditions in the medium. In both low and high glucose lipolytic conditions, the highest rate of glycerol release was observed between *T* = 0–15 h, with progressively slower rates of release between *T* = 15–39 h and *T* = 39–63 h (Figure 4). Those patterns were observed for both inner and outer blubber, as well as for early and late fasting pups. Moreover, when PCATS maintained in culture under non-lipolytic conditions for 39 h were stimulated to induce lipolysis, there was a significant increase in glycerol release (*p* < 0.05) (Figure 4).

**FIGURE 5:**
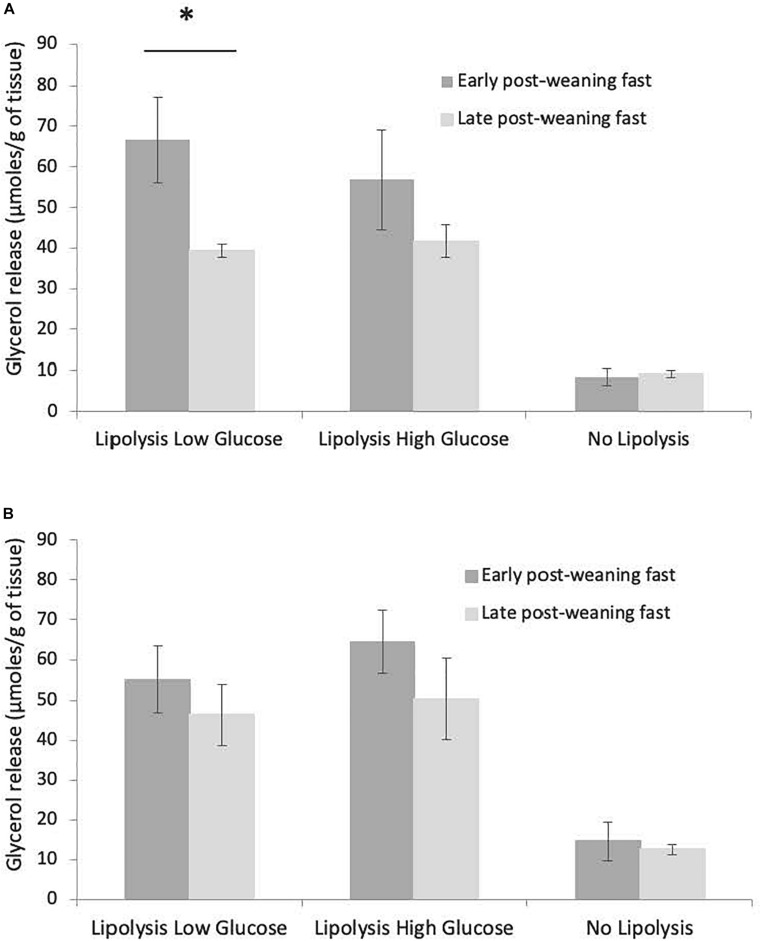
Comparison of total glycerol released between early and late weaned northern elephant seal pups, for inner **(A)** and outer **(B)** blubber and the three tested treatments (“Lipolysis low Glucose,” “Lipolysis High Glucose,” “No Lipolysis”). Glycerol concentrations were normalized per unit of initial wet weight (before starting the culture). Results are expressed as mean ± SEM of six pups at early fast and mean ± SEM of eight pups at late fast. Differences were tested using Student *t*-test (non-paired data). “^∗^” refers to values significantly different (*p* < 0.05) between early and late post-weaning fast within a particular treatment.

The total release of glycerol over 63 h of culture in the three conditions (LLG, LHG, NL) did not vary with sex or blubber depth (inner or outer blubber), either at early or late fast (data not shown). In inner blubber, the total release of glycerol was significantly higher at early as compared to late fast in the LLG condition (*p* < 0.05), whereas it did not change for the other conditions (Figure 5A). In outer blubber, the fasting stage (early or late fast) did not affect the total release of glycerol in culture media for the three conditions investigated (Figure 5B).

### Leptin Secretion

**FIGURE 6:**
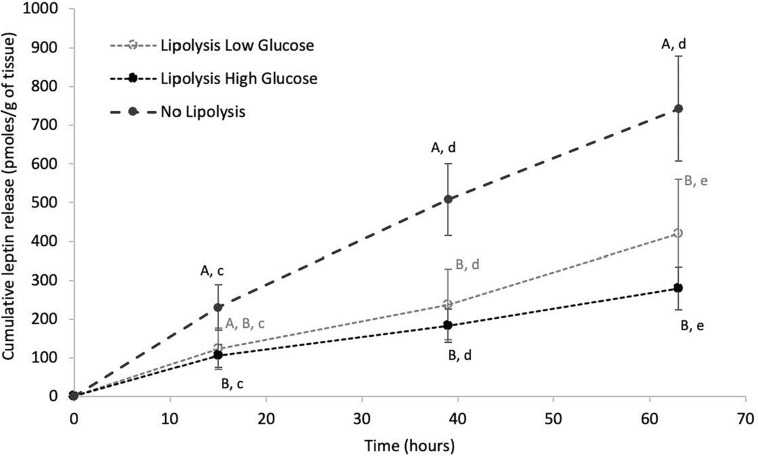
Cumulative leptin release over time from inner blubber slices of early weaned northern elephant seal pups in “Lipolysis Low Glucose,” “Lipolysis High Glucose” and “No Lipolysis” conditions. Leptin concentrations were normalized per unit of initial tissue wet weight (before starting the culture). Results are expressed as mean of four pups ± SEM. Differences between treatments within a particular time or over time within a particular treatment were assessed with linear mixed models and Tukey’s tests. Values with no common capital letter A, B, C are significantly different (*p* < 0.05) within a precise time between treatments. Values with different lowercase letters d, e, f are significantly different (*p* < 0.05) within a precise treatment over time.

Leptin release over time in culture media was analyzed in inner and outer blubber and at early and late fast in the NL, LLG and LHG conditions. Cumulative leptin release in culture media increased significantly over time in NL, LLG and LHG conditions, especially during the first 39 h of culture (*p* < 0.05) (Figure 6). At early fast, the rate of leptin secretion by inner blubber slices was higher in the NL condition. As a result, the total leptin production over 63 h was significantly higher in the NL condition as compared to the two lipolytic conditions (*p* < 0.05), the latter of which did not differ from one another (Figure 6). The situation was slightly different for outer blubber slices, where the leptin produced in the NL condition significantly exceeded the one in the LHG condition (*p* < 0.05) but not the one in the LLG condition. At late fast, the difference of leptin production between the NL and the lipolytic conditions (factor 1.5–2) was not significant (inner or outer blubber).

**FIGURE 7:**
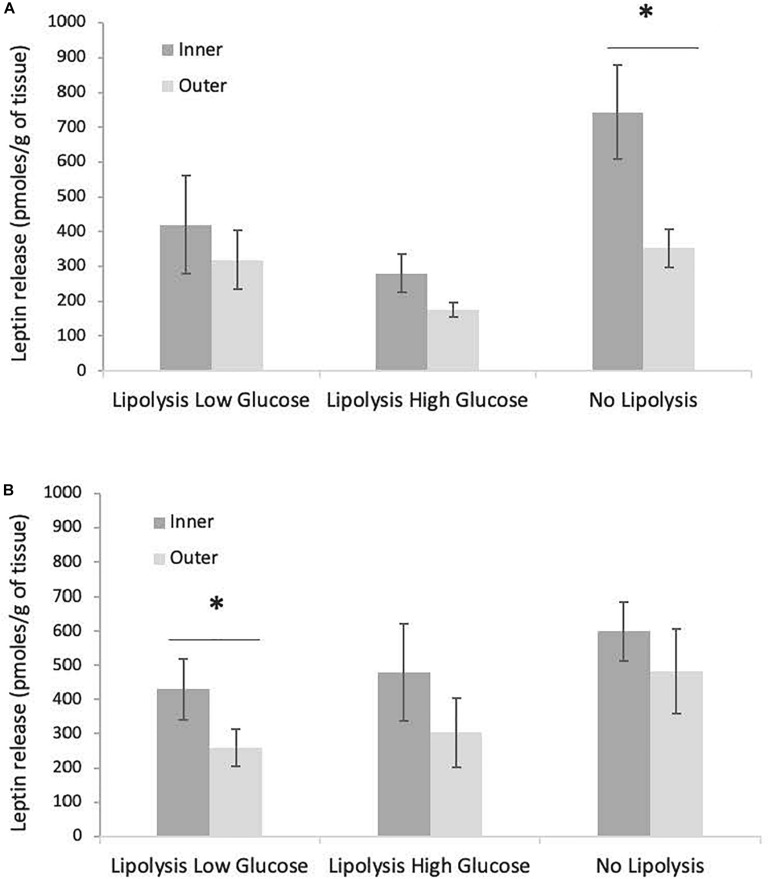
Comparison of total leptin released, between inner and outer blubber, for early **(A)** and late **(B)** weaned pups and three tested treatments (“Lipolysis Low Glucose,” “Lipolysis High Glucose,” and “No Lipolysis”). Leptin concentrations were normalized per unit of initial wet weight (before starting the culture). Results are expressed as mean ± SEM of four pups at early fast and mean ± SEM of six pups at late fast. Differences were tested using Student *t*-test (paired data). ^∗^Refers to values significantly different (*p* < 0.05) between inner and outer blubber within a particular treatment.

The total release of leptin over 63 h of culture in the three conditions (LLG, LHG, NL) did not vary with sex or fasting stage (data not shown). In contrast, the quantity of leptin released by inner blubber slices tended to be higher than the amount released by outer blubber slices. This difference was however only significant for the NL condition at early fast, where a 50% drop of leptin production could be observed between inner and outer blubber, and for the LLG condition at late fast (*p* < 0.05) (Figures 7A,B).

## Discussion

In the present study, we developed a PCATS model from NES. A notable and compelling advantage of PCATS, as compared to tissue explants more generally, is that they generate samples of standardized and reproducible physical dimensions with comparable diffusion of oxygen and nutrients, thus reducing variation in these factors in comparisons across experimental treatments. Since NES adipose tissue is soft and difficult to secure during slicing, it was important to find a device in which the tissue biopsy could be anchored before slicing. We therefore chose a compact device designed for making thin slices of rat spinal cord, as it contains a groove of approximately the same width as the NES blubber biopsies (Figure 1). The diameter of the slices obtained here and the ratio cell size/slice thickness were within the range usually recommended for precision-cut slices of other tissues. Indeed, in general, precision-cut tissue slices should be thin enough to allow the diffusion of substrates present in the medium into the innermost cell layers. They also have to be thick enough to preserve the natural architecture of the tissue and minimize the proportion of damaged cells (on the cutting edges) (de Kanter et al., 2005; Fisher and Vickers, 2013). Within the few publications preparing thin slices of adipose tissue, the best results regarding metabolic activity of the slices, based on conversion of ^14^C glucose into lipids (Faulhaber et al., 1972; Ramsay and Rosebrough, 2005), expression of lipid droplet surface protein by immunofluorescence (Schopow et al., 2020) as well as morphological investigations (Anayama et al., 2015), were observed with slices thickness of 400 to 1,000 μm. The thickness of the slices obtained here falls within this range. NES PCATS exhibited good viability over at least 63 h in culture, which is in accordance with the viability reported for precision-cut slices of other tissues (Thiele et al., 2017). Most of the publications on adipose tissue slices report a good viability up to 24 h of culture (Mersmann and Shparber, 1993; Ramsay and Rosebrough, 2005; Cai et al., 2011). Only two of them cultured thin adipose tissue slices for longer periods, i.e., five (Anayama et al., 2015) and 14 days (Schopow et al., 2020).

**FIGURE 8:**
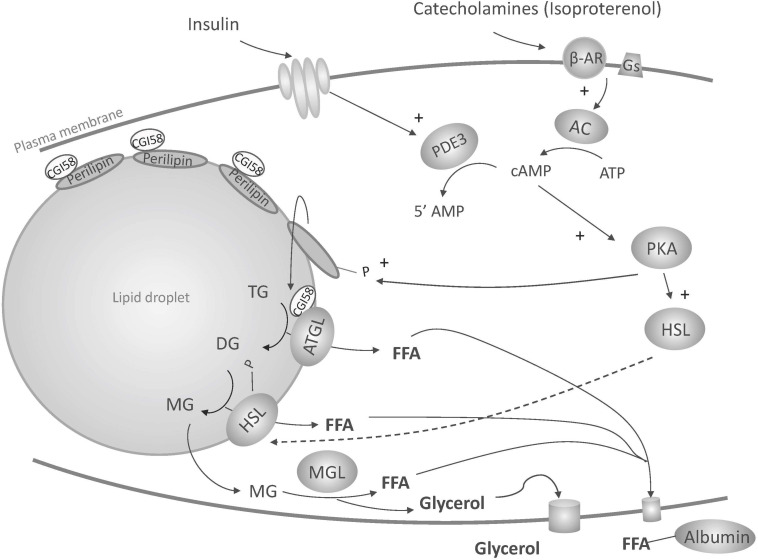
Illustration of lipolysis induced by ß-adrenergic stimulation. ß-AR, ß-adrenergic receptor; Gs, stimulatory guanosine triphosphate (GTP)-binding proteins; AC, adenylate cyclase; cAMP, cyclic adenosine monophosphate; ATGL, adipose triglyceride lipase; ATP, adenosine triphosphate; CGl-58, comparative gene identification-58; DG, diglyceride; FFA, free fatty acid; HSL, hormone sensitive lipase; MG, monoglyceride; MGL, monoglyceride lipase; P, phosphate; PDE3, phosphodiesterase 3; PKA, protein kinase A; TG, triglyceride; 5’AMP, 5’ adenosine monophosphate. See text for more details.

Metabolic function of NES PCATS was assessed through lipolysis, an elementary metabolic function of adipose tissue. During this catabolic pathway, the energy stored in adipocytes as triglycerides is released through the breakdown of triglycerides into glycerol and free fatty acids. This process occurs in three steps, triglycerides being first hydrolyzed into diglycerides, then monoglycerides and then glycerol. Each step leads to the release of one fatty acid molecule, under the sequential activity of three lipases: adipose triglyceride lipase (ATGL), hormone sensitive lipase (HSL) and monoglyceride lipase (MGL) (Figure 8). Among other signals, catecholamines can trigger lipolysis through their binding to ß-adrenergic receptors that leads to the activation of adenylate cyclase and the subsequent increase in cytosolic cyclic adenosine monophosphate (cAMP), which in turn activates protein kinase A (PKA). PKA promotes the phosphorylation of HSL and perilipin A. Phosphorylation of HSL leads to its activation and migration from the cytosol to the surface of the lipid droplet. The phosphorylation of perilipins, which initially form a coat around the droplets to prevent access of enzymes, promotes access of ATGL and HSL to the lipid droplet in addition to the release of CGI-58 (comparative gene identification-58, also known as ABHD5 for α/β hydrolase domain-containing 5), which is sequestered by perilipins under unstimulated conditions. Its release into the cytoplasm and subsequent interaction with ATGL activates triglyceride hydrolysis (Granneman et al., 2007, 2009) (Figure 8). Free fatty acids generated are released in the blood circulation. They are then transported by albumin to target tissues such as the liver and muscle where they are ß-oxidized. Some free fatty acids can also be reabsorbed by the adipocyte itself or by neighboring adipocytes to resynthesize triglycerides (Zimmermann et al., 2004). Glycerol generated during lipolysis will be poorly used by the adipocytes since glycerol kinase activity is very low in adipose tissue. Instead, it will be released in the blood circulation. Glycerol is thus a useful indicator of *in vitro* lipolytic activity as, contrary to fatty acids, it is not recycled by adipocytes.

In the present study, the NES PCATS remained functional throughout the culture, as lipolysis could be efficiently triggered on tissues that had been in the non-lipolytic condition for the first 39 h of culture. Such results are in accordance with the viability results. Lipolysis was triggered with isoproterenol, a synthetic agonist of ß-adrenergic receptors, in the presence of albumin, as often reported in rodent and human *in vitro* lipolysis protocols (Ottosson et al., 2000; Harmancey et al., 2005; Campbell et al., 2011; Louis et al., 2014d). Albumin binds fatty acids resulting from lipolysis (Figure 8). In cultured human adipocytes, its presence in the culture medium was proven to be essential to induce a significant increase of glycerol in the culture medium after ß-adrenergic stimulation (Burns et al., 1975). A similar phenomenon was also observed for NES PCATS (C. Debier, unpublished results). The presence of albumin in the culture medium is indeed important to prevent an intracellular accumulation of fatty acids that could otherwise inhibit adenylate cyclase activity (Mottillo and Granneman, 2011). Insulin is a main antilipolytic agent. The binding to its receptor induces a cascade of reactions that finally lead to the degradation of cAMP into 5′AMP, which negatively modulates lipolysis by reducing HSL, ATGL and perilipins activation (Figure 8). In order to keep the antilipolytic action of insulin as low as possible on the LLG and LHG conditions, the insulin concentrations were 10 times lower than in the NL condition. The insulin concentrations used in lipolytic and non-lipolytic conditions were respectively slightly below and slightly above the range of concentrations encountered *in vivo* in NES pups (34.8 to 94.1 pg/mL) (Houser et al., 2013). Insulin circulating levels are generally low in NES and insulin sensitivity declines over the fast, allowing seals to sustain long-term lipolysis as well as glucose and ketone body availabilities to specific tissues with limited metabolism of lipids (Houser et al., 2007; Viscarra et al., 2012; Crocker et al., 2014). NES exhibit high circulating glucose concentrations during the fast (Houser et al., 2013; Crocker et al., 2017), the highest concentrations being encountered in fasting pups (around 9 mM or 1.62 g/L). The final glucose concentrations in our LHG and LLG conditions were respectively 2.6 times higher and 1.8 times lower than the glucose concentrations encountered *in vivo* in NES pups (Houser et al., 2013). The level of glucose did not influence lipolysis efficiency. Yet, *in vitro* studies on rodent and human adipose tissue showed that glucose can help to maintain lipolysis. However, such results were observed on a shorter time frame (1–2 h) (Knight and Iliffe, 1973; Naito and Okada, 1975) and/or with lower glucose concentrations (Knight and Iliffe, 1973; Smith and Jacobsson, 1973). The stimulating effect of glucose on lipolysis was also usually observed when insulin was added to the culture medium (which was not the case in our lipolytic conditions), suggesting that glucose can overcome the antilipolytic effect of insulin (Knight and Iliffe, 1973).

The only studies reporting *in vitro* culture of adipose tissue explants of marine mammals were conducted using gray seal blubber (Bennett et al., 2017; Robinson et al., 2018). Several technical distinctions can be observed with the present study. First, adipose tissue was randomly minced instead of being precisely cut. Second, gray seal explants were cultured as 100 mg per condition (i.e., 10 pieces of around 5–10 mg each), in 1 mL of culture medium without agitation. In the present study, four slices of 4–5 mg of NES adipose tissue were cultured in 1 mL of culture medium with agitation. The NES PCATS may therefore have had a better and more reproducible oxygenation and contact with medium components. Gray seal blubber explants were also cultured over a shorter period of time (24 h instead of 63 h in the present study). In Bennett et al. (2017), the two different glucose concentrations in culture media corresponded to levels used in the present study. No ß-adrenergic agonist or albumin were added in the culture medium. Conditions were therefore closer to the non-lipolytic (NL) treatment of the present study. The levels of glycerol released over 24 h were indeed comparable to the levels released in the NL condition and much lower than the levels released in the LLG and LHG conditions.

*In vivo*, inner and outer blubber layers in marine mammals appear to differ in terms of lipid composition and mobilization, as well as pollutant load (Best et al., 2003; Debier et al., 2003, 2006; Strandberg et al., 2008; Louis et al., 2014a, 2015). Inner blubber is usually considered as more metabolically active and is the first layer to be mobilized during negative energy balance while outer blubber is more dedicated to insulation and buoyancy. The differences in lipid and pollutant mobilization observed *in vivo* between inner and outer blubber could be due to differences of vascularization between the two layers. We did not observe any significant difference of *in vitro* glycerol release following ß-adrenergic stimulation between slices from inner and outer blubber. The fact that, contrary to the *in vivo* situation, the slices resulting from the two blubber layers were exposed to the exact same lipolytic conditions (LLG, LHG) may explain why they exhibited the same response. Louis et al. (2015) showed that there is no significant difference of ATGL and HSL gene expression between inner and outer blubber of weaned NES pups, either at early or late fast, which supports the results observed here.

*In vivo*, circulating free fatty acid and glycerol levels of NES increase over the fast while the lipolytic rate appears to remain constant (Houser et al., 2007; Crocker et al., 2014). ATGL and HSL expressions do not significantly change over the pup post-weaning fast in either inner or outer blubber (Louis et al., 2015). However, at the protein level, ATGL appears to increase whereas HSL remains constant (Viscarra et al., 2012) or decreases (Viscarra and Ortiz, 2013; Viscarra et al., 2013) over the fast in NES pups, reflecting potential post-transcriptional regulations. Yet, differences between inner and outer blubber were not investigated in those studies. The increase of adipose ATGL protein levels combined to the decrease in HSL levels and the consistent rise in plasma NEFA:glycerol ratios suggest that diglyceride hydrolysis is reduced, preventing premature depletion of lipid stores (Viscarra and Ortiz, 2013). In the present study, the *in vitro* tissue response to isoproterenol was significantly higher at early as compared to late fast, in inner blubber slices from the LLG condition. A tendency toward a higher release of glycerol at early compared to late fast in inner blubber could also be observed in the LHG condition, but the difference was however not significant. This lower amount of glycerol release following ß-adrenergic stimulation could result from incomplete triglyceride hydrolysis in late fasting slices as observed *in vivo*. The absence of variation between early and late fast in outer blubber likely reflects a differential evolution of the two blubber layers over the fast. In Robinson et al. (2018), glycerol release was higher in explants from feeding gray seal pups as compared to fasting gray seal pups. The authors explain this difference by the higher metabolic rate of the tissue coming from animals in a feeding state. The decreasing metabolic rate between early and late fast could also explain the results observed here.

Metabolic function of NES PCATS was also assessed through leptin production, which has never been investigated *in vitro* for marine mammal adipose tissue. This adipokine is considered as one of the most important energy regulating hormones. NES PCATS secreted leptin *in vitro.* Its release in the culture medium was however dependent on several factors, such as the culture condition. Indeed, the addition of isoproterenol significantly dropped leptin discharge in the culture medium, especially in inner blubber from early fast. The negative impact of ß-adrenergic stimulation on leptin levels is in accordance with what is observed for other mammals such as rodents (Caron et al., 2018). The exact mechanism through which the activation of ß-adrenergic receptors regulates leptin expression is however not known (Caron et al., 2018). Fasting is known to be negatively correlated to leptin production. Circulating leptin levels significantly dropped between early and late fast in NES adult males (Crocker et al., 2012) as well as in adult bowhead whales (*Balaena mysticetus*) between feeding and fasting periods of their life cycle (Ball et al., 2017). In the present study, there was however no impact of fasting stage on *in vitro* leptin production by the tissue slices, which is in accordance with the results obtained on NES adult females and weaned pups (Viscarra et al., 2012; Khudyakov et al., 2019).

The tissue layer had a significant effect on leptin production, as inner blubber slices exhibited higher leptin release in the culture media as compared to outer blubber slices, especially in the non-lipolytic condition from early fast. This trend may result from the fact that inner blubber is more metabolically active than outer blubber. The differential synthesis of leptin as a function of blubber depth was also observed in bowhead whales, where leptin transcripts were higher in inner blubber as compared to outer blubber in well-fed adults (Ball et al., 2017). The opposite tendency was however observed in fasting individuals.

To summarize, we have developed the first PCATS model for a marine mammal species, using a portable device that is compact, cheap and easy to use. Our results show that slices precisely cut into 1 mm thickness remain alive and functional in culture at 37°C for at least 63 h. Lipolysis, a fundamental metabolic function of adipose tissue, was investigated to assess the biological function of tissues in culture. The slices exhibited an efficient response to ß-adrenergic stimulation even after 2 days of culture. *In vitro* lipolysis efficiency did not vary between outer and inner blubber but was significantly higher at early as compared to late fast for slices of the inner blubber layer. At early fast, the production of leptin by the slices was higher in the non-lipolytic condition. Inner blubber slices were also more efficient at producing leptin than outer blubber slices. This model of PCATS has been established on a marine mammal model that is easily accessible. It represents a useful tool to increase the mechanistic understanding of the effects of environmental and physiological stressors, with future applicability to other marine mammal species, including large cetaceans.

## Data Availability Statement

The data generated for this study are available on request to the corresponding author.

## Ethics Statement

The animal study was reviewed and approved by the Sonoma State University Institutional Animal Care and Use Committee (IACUC #2017-56).

## Author Contributions

CaD conceived the study and designed field sampling, tissue culture and related experiments with input from DC, LP, ClD, and J-FR. CaD and MV carried out sample collection, tissue cultures and associated biochemical tests. DC supervised sample collection and leptin analyses. CR performed leptin assays. LP and GT conducted data analyses. Tissue culture experiments were carried out in DS’s lab, with all the access to cell culture facilities. CaD and DC were involved in funding acquisition. CaD drafted the manuscript with input from LP and MV, DC, J-FR, DS, YL and GT edited, revised and approved the final version of the manuscript. All authors contributed to the article and approved the submitted version.

## Conflict of Interest

The authors declare that the research was conducted in the absence of any commercial or financial relationships that could be construed as a potential conflict of interest.
